# The analysis of the pyroptosis-related genes and hub gene TP63 ceRNA axis in osteosarcoma

**DOI:** 10.3389/fimmu.2022.974916

**Published:** 2022-11-01

**Authors:** Jun Han, Yunxiang Hu, Shengqiang Ding, Sanmao Liu, Hong Wang

**Affiliations:** ^1^ School of Graduates, Dalian Medical University, Dalian, China; ^2^ Department of Orthopedics, Dalian Municipal Central Hospital, Dalian City, China; ^3^ Department of Spine Surgery, The People’s Hospital of Liuyang City, Changsha, China

**Keywords:** pyroptosis, osteosarcoma, TP63, ceRNA, immunotherapy, tumor microenvironment

## Abstract

Pyroptosis is a type of programmed cell death that is associated with tumor development, prognosis, and therapeutic response. The significance of pyroptosis-related genes (PRGs) in the tumor microenvironment (TME) remains unclear. We examined the expression patterns of PRGs in 141 OS samples from two different datasets and characterized the genetic and transcriptional changes in PRGs. Based on these PRGs, all OS samples could be classified into two clusters. We discovered that multilayer PRG changes were linked to clinicopathological traits, prognosis, and TME characteristics in two separate genetic subtypes. The PRG score was then developed for predicting overall survival, and its predictive efficacy in OS patients was tested. As a result, we developed a very precise nomogram to improve the PRG-predictive model in clinical application. Furthermore, a competing endogenous RNA (ceRNA) network was built to find a LAMTOR5-AS1/hsa-miR-23a-3p/TP63 regulatory axis. Through experimental verification, it was found that the pyroptosis gene TP63 plays an important role in the regulation of osteosarcoma pyroptosis. The possible functions of PRGs in the TME, clinicopathological characteristics, and prognosis were established in our investigation of PRGs in OS. These findings may aid in our understanding of PRGs in OS as well as provide a novel way for prognostic evaluation and the creation of more effective immunotherapy treatments.

## Introduction

Osteosarcoma (OS) is the most common primary malignant bone tumor in children and young adults, usually occurring in the metaphysis of long bones (1). Patients with localized tumor present a 5-year survival rate of 60%, while those with metastatic tumor have a 5-year survival rate of only 20% (2). Despite the current standard treatment for primary bone osteosarcoma, which consists of neoadjuvant chemotherapy and surgery, the survival rate has significantly improved, and its treatment outcomes are unfavorable owing to tumor invasion and metastasis (3). Therefore, defining a novel prognostic gene markers model of OS is imperative for improving the overall survival of OS patients.

Pyroptosis is a type of programmed cell death caused by inflammation, which is unusual compared to other kinds of programmed cell death (4). The cleavage of the gasdermin family characterizes pyroptotic cells through classical pathways, non-classical pathways, the caspase-3/8-mediated pathway, and the granzyme-mediated pathway, followed by the cell membrane ruptures and the release of the cell contents (5). Many studies indicated that pyroptosis plays a pivotal role in the pathogenesis and progression of multiple cancers. However, pyroptosis is complicated in cancers and exhibit cancer-inhibiting or cancer-promoting activities in different cancers (6, 7). Previous studies also indicate that there are associations between pyroptosis and the tumor microenvironment (TME) (8, 9). Especially, a wide variety of immune cell types are involved in the TME, primarily lymphocytes, dendritic cells, macrophages, mast cells, neutrophils, and myeloid-derived suppressor cells (10, 11). These cells can inhibit tumor progression by recognizing and killing tumor cells. Thus, immunotherapy has emerged as an effective therapeutic approach to killing tumor cells by activating immune responses (12, 13). However, as compared to other cancers, there have been fewer investigations into immunotherapy for OS. Hence, a comprehensive analysis of the TME mediated by pyroptosis-related genes (PRGs) may be more helpful to understand the underlying mechanism of OS tumorigenesis and guide clinical therapy.

We used the RNA sequencing data of OS patients and normal muscle-skeletal tissues downloaded from the Therapeutically Applicable Research to Generate Effective Treatments (TARGET) and Genotype-Tissue Expression (GTEx) databases to construct a tumor vs. normal datasets for identifying differentially expressed PRGs (DEPRGs). We identified two pyroptosis-related subtypes of OS according to DEPRGs. In addition, two advanced computational algorithms gave us a comprehensive view of the immune cell infiltration landscape of OS: the Cell-type Identification By Estimating Relative Subsets Of RNA Transcripts (CIBERSORT) and Estimation of Stromal and Immune cells in Malignant Tumor tissues using Expression data (ESTIMATE). Furthermore, we constructed a five-gene signature (PRG_score) by using the LASSO–Cox method to predict prognosis, immune infiltration, and chemotherapy drugs. Lastly, we constructed a PRG competing endogenous RNA (ceRNA) network and found one hub gene in the pyroptosis regulation of OS cells.

## Materials and methods

### OS data source and preprocessing

The RNA sequencing data, clinical information, and copy number variation (CNV) data of osteosarcoma patients were downloaded from the TARGET-OS database (https://xenabrowser.net/datapages/), and the RNA sequencing data of 396 normal human muscle-skeletal tissue samples were downloaded from the GTEx database (https://xenabrowser.net/datapages/). Two datasets are fragments per kilobase million (FPKM) value, and the expression data were normalized to log2 (FPKM + 1) before merging the two datasets. The microarray datasets of GSE21257 (53 OS patients) were downloaded from the Gene Expression Omnibus (GEO) database (https://www.ncbi.nlm.nih.gov/geo/). Because the expression profile data of the TARGET dataset (FPKM value) were significantly different from the microarray data (transcripts per kilobase million, TPM), we transformed the TARGET data into TPMs by the “limma” R package. Then, we merged TARGET and GEO into a dataset including 141 OS patients. The “combat” algorithm of the “sva” package was applied to address the batch effects caused by non-biological technical biases. Further analysis was not conducted on patients without survival information.

### Identification of DEPRGs and consensus clustering analysis

A total of 52 PRGs were retrieved from the MSigDB Team (REACTOME_PYROPTOSIS) (http://www.broad.mit.edu/gsea/msigdb/) and prior reviews, which are shown in **Table S1** (6, 14–17). The “limma” package was used to determined DEPRGs by setting the cutoff criteria as p-value <0.05. After merging the RNA expression of the TARGET cohort and GEO cohort into a dataset with 141 OS patients, consensus clustering analysis was performed to identify distinct pyroptosis patterns based on the expression of PRGs and cluster the 141 OS patients for further analysis. The number of PRGclusters and their stability were determined by increasing the “k” index from 2 to 9 using the R package “ConsensuClusterPlus.”

### Functional enrichment analyses

To study the differences in PRGcluster in biological processes, the “GSVA,” “limma,” and “pheatmap” R packages were used to perform enrichment analysis in a heatmap with the hallmark gene set (c2.cp.kegg.v7.2) downloaded from the MSigDB database (https://www.gsea-msigdb.org). The single-sample gene set enrichment analysis (ssGSEA) has been conducted using the R package “GSVA” to calculate the scores of infiltrating immune cells. We identified DEGs between PRGclusters using the “limma” package. The Gene Ontology (GO) and Kyoto Encyclopedia of Genes and Genomes (KEGG) analyses were performed by applying the “clusterProfiler” package based on the DEGs between PRGclusters, with p-value <0.05.

### Immune infiltration analysis

CIBERSORT was applied to estimate the relative abundance of 22 tumor-infiltrating immune cell subtypes in each sample of the TARGET and GEO cohorts using the R package. The ESTIMATE algorithm was exploited to determine the fractions of stromal and immune cells in tumor samples of the TARGET and GEO cohorts using the “estimate” R package.

### The establishment of the pyroptosis score model and prognostic analysis

The pyroptosis score system was established to quantify the pyroptosis patterns of the OS patients. The method of constructing the pyroptosis score system is as follows: the DEGs of different PRGclusters were subjected to univariate Cox regression analysis where p-value <0.05 was considered statistically significant. The TARGET and GEO cohorts were randomly divided into training set and testing set with a proportion of 1:1 by using the “caret” package. After that, by using Least Absolute Shrinkage and Selection Operator (LASSO) regression, we were able to further compress the screened genes and eventually identified a novel gene signature. Using the LASSO regression results, we developed a prognostic risk score formula, which was calculated as follows: Risk score = patient × i Coefficient (mRNAi) × Expression (mRNAi). The training set, testing set, and all sets were classified into low and high PRG-score groups. The efficiency of the model was determined by the Kaplan–Meier method and time-dependent receiver operating characteristic (ROC) curve constructed with the “SurvivalROC” package. The clinical characteristics (gender, age, and metastasis) of patients were extracted from the TARGET cohort and the GEO cohort to construct a nomogram to predict the overall survival of OS patients after 1, 3, and 5 years.

### PRG competing endogenous RNA network construction

Different-expression pyroptosis-related mRNAs (DEPRMs), different-expression miRNAs (DEMis), and different-expression lncRNAs (DELs) between the TARGET samples and matching GTEx normal samples were identified using the limma package. The adjusted p-value of DEMs, DEMis, and DELs was defined as <0.05, and the log2 fold changes (|log2FC|) of DEMs and DELs were defined as >1 and 2, respectively. The weighted gene co-expression network analysis (WGCNA) package in R software was used to create gene co-expression networks based on DEMs, DEMis, and DELs. First, outliers in samples with low expression data were identified and eliminated. Following that, the mean connectivity and scale-free fit index for numbers 1–30 [as soft-threshold power (β)] were determined individually, with the best result determining the adjacency matrix’s co-expression similarity. The estimated correlation matrix (based on Pearson’s correlation) was then transformed to an adjacency matrix, and a topological overlap matrix (TOM) was constructed, which takes into account indirect gene interactions. The negative interactions of miRNA–mRNA and miRNA–lncRNA were used to make the lncRNA–miRNA–mRNA network, which was constructed by using Cytoscape 3.5.1 (www.cytoscape.org/) based on co-expression WGCNA data. The Cytoscape “plugin molecular complex detection” (MCODE) was used to find the most relevant subnetworks, using the following cutoff value: node score cutoff = 0.2, degree cutoff = 2, max depth = 100, and k-core = 2. To construct a PRG ceRNA network, the starBase database (starBase, v2.0, http://starbase.sysu.edu.cn/) was further applied to identify the potential relationship of lncRNA–miRNA–mRNA.

### Cell culture and transfection

The ScienCell Research Laboratories (USA) provided two OS cell lines (143B and U2OS). Cell lines were cultured at 37°C in Dulbecco’s modified Eagle’s medium (Gibco, USA) supplemented with 10% (v/v) fetal bovine serum (Invitrogen, USA) in a 5% CO_2_ atmosphere. The four types of pGPU6/GFP/Neo vector shRNA targeting TP63 and the three types of Lamtor-AS1 siRNA (GenePharma, Shanghai, China) were transfected by Lipo3000 (Invitrogen, USA) according to the manufacturer’s protocol. Sequences of siRNA and shRNA are shown in **Table S2**. The experiments were implemented in three groups as follows: the knockdown group (cells transfected with siRNA or shRNA), the NC group (cells transfected with NC), and the control group (untransfected cells).

### Quantitative real-time PCR

Quantitative real-time PCR (qRT-PCR) was used to determine the relatively higher knockdown efficiency of shRNA and siRNA for further experiments. Total RNA was extracted from OS cells using TRIpure Reagent (Bioteke, Beijing, China). The BeyoRT II M-MLV Reverse Transcriptase (Beyotime Biotechnology, Shanghai, China) and RNase inhibitor (Bioteke, Beijing, China) were used for reverse transcription. The 2×Taq PCR MasterMix and SYBR Green (Solarbio, Beijing, China) were employed to carry out the qRT-PCR assay. In order to normalize lncRNA and mRNA expression, β-actin was used as an endogenous control. 2^−ΔΔCt^ was used to calculate the relative expression level of the target RNA. **Table S3** lists the primers used for target RNA amplification.

### Western blotting

The cells were harvested in RIPA Lysis Buffer and lysed using ultrasound (Wanleibio, Shenyang, China). BCA Reagent was used to determine total protein content (Wanleibio, Shenyang, China). SDS-PAGE (Wanleibio, Shenyang, China) was used to separate equivalent quantities of protein extract, which was then deposited onto PVDF membranes (Millipore, USA). Cleaved-Caspase-1 (Wanleibio, Shenyang, China), cleaved-Caspase-3 (Wanleibio, Shenyang, China), cleaved-Caspase-4 (Affinity Biosciences, Suzhou, China), cleaved-Caspase-8 (Affinity Biosciences, Suzhou, China), GSDMD (Affinity Biosciences, Suzhou, China), GSDME (ABclonal, Wuhan, China), and GSDMD-N (Affinity Biosciences, Suzhou, China) were the primary antibodies employed in this test. After blocking with 5% skim milk for an hour, the membranes were incubated overnight with primary antibodies at 4°C. The membranes were then incubated with HRP-conjugated secondary antibodies (Wanleibio, Shenyang, China) and detected using an enhanced chemiluminescence substrate kit (Wanleibio, Shenyang, China) after washing.

### Statistical analysis

Statistical comparisons between groups were made using the Student’s *t*-test. Data were provided as mean and standard deviation. Statistical significance was defined as a p value of less than 0.05. SPSS V. 26.0 (IBM, NY, USA) was used to conduct all statistical tests.

## Results

### Genetic variation and expression of PRGs in OS

**Figure 1:**
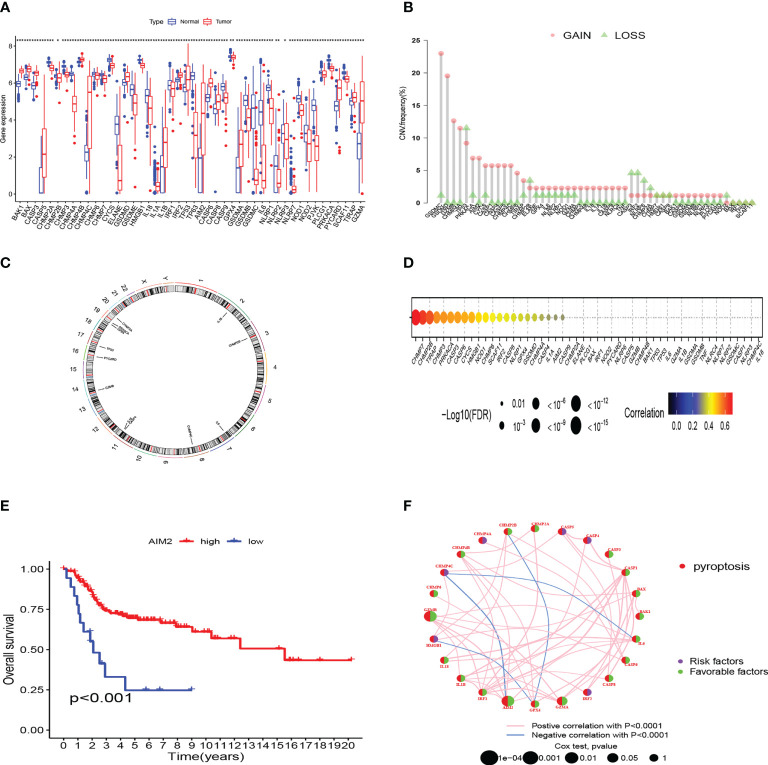
Genetic and transcriptional characteristics of PRGs in OS. **(A)** The differential expression of 46 PRGs between normal and OS tissues. (***:p value <0.001, **:p value <0.01, *:p value <0.05) **(B)** CNV frequency of PRGs in the TARGET cohort. **(C)** Locations of CNV alterations of PRGs on 23 chromosomes by the TARGET cohort. **(D)** The correlation analysis between CNV of PRGs and RNA sequence expression of PRGs in the TARGET cohort. **(E)** The K-M curves of AIM2 gene in OS. **(F)** Prognosis value and correlations between PRGs in OS. The line linking the PRGs is their correlation. PRGs, pyroptosis-related genes; OS, osteosarcoma; CNV, copy number variations.

We compared the 52 PRG expression levels between human OS samples (TARGET) and normal muscle-skeletal tissues (GTEx) and found that 46 PRGs expressed differently (p-value < 0.05) (**Figure 1A**). To evaluate the levels of CNV among OS patients, we analyzed the CNV data from TARGET. **Figure 1B** shows that CHMP4A, GSDMD, GZMB, and GSDMC represented the highest frequency of CNV gain and TP53, CHMP2B, CASP3, and IRF2 represented the highest frequency of CNV loss. Also, we located the 12 PRGs with CNVs on their respective chromosomes (**Figure 1C**). In the correlation analysis between PRG CNV and PRG RNA sequence expression, CHMP7, CHMP2B, TIRAP, and CHMP3 had the strongest correlation with their CNV (**Figure 1D**). After integrating the data of survival time and gene expression of the TARGET cohort and GEO cohort, the expressions of 28 PRGs were obtained from 141 patients (**Table S4**). We performed Kaplan–Meier (K–M) survival curve analysis on the PRGs, and the results indicated that the most abnormal expression of PRGs was significantly related to the prognosis of OS patients. The high expression of CASP5, CHMP4A, CHMP4C, and HMGB1 correlated with patients’ poor prognosis. The high expression of AIM2, BAK1, CASP1, CASP6, CHMP2A, CHMP4B, CHMP6, CHMP7, GPX4, GZMA, GZMB, and IL1B correlated with patients’ better prognosis (**Figure 1E**). The comprehensive landscape of PRG correlation and prognostic value in patients with OS was demonstrated in a prognosis network by a univariate Cox regression analysis and co-expression analysis (**Figure 1F**). The results were consistent with the K–M survival analysis showing that CASP4, CASP5, CHMP4A, CHMP4C, HMGB1, and IRF2 were risk factors for OS patients.

### Identification of pyroptosis clusters mediated by 28 pyroptosis-related regulators

**Figure 2:**
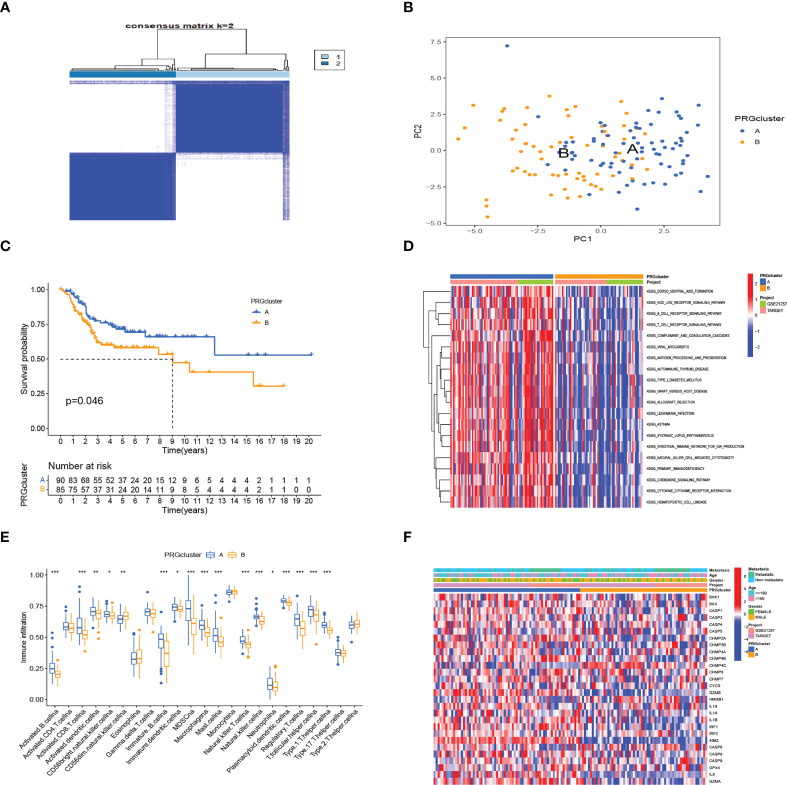
Subtypes of OS divided by pyroptosis-related regulators. **(A)** Consensus matrix heatmap defining two clusters (k = 2) in the TARGET and GSE21257 cohorts. **(B)** PCA of the expression of PRGs indicating a significant difference between the two clusters. **(C)** Kaplan–Meier analysis showing the survival of the two clusters. **(D)** Differences in characteristics of clinicopathology and PRG expression levels among two clusters. **(E)** In the two subtypes, the tumor infiltration of 24 immune cell types. (***:p value <0.001, **:p value <0.01, *:p value <0.05) **(F)** The heatmap was used to depict the active biological pathways in different pyroptosis-related clusters, which were examined by GSVA. OS, osteosarcoma; PRGs, pyroptosis-related genes.

We obtained 28 PRG expression levels of the cohort consisting of two OS datasets (TARGET, GEO). Based on the 28 PRG expression levels, two different OS patterns were determined by using the unsupervised clustering method (k = 2), including 76 cases in PRGcluster A and 65 cases in PRGcluster B (**Figure 2A**). The two-dimensional principal component analysis (PCA) biplots showed significant differences between the pyroptosis transcription profiles of the two subtypes (**Figure 2B**). The K–M curve revealed that the overall survival rate of PRGcluster A is better than that of PRGcluster B (p-value < 0.05) (**Figure 2C**). There is no significant difference in the clinicopathological features of these two different clusters (**Figure 2D**). The ssGSEA algorithm was employed to estimate the relative infiltration of 24 intratumoral immune cell types for 141 OS samples. We found that PRGcluster A was remarkably richer in the infiltration of most immune cells than PRGcluster B. The infiltration levels of B cell, CD8 T cell, dendritic cell, MDSC, macrophage, mast cell, killer T cell, natural killer cell, plasmacytoid dendritic cell, regulatory T cell, T follicular helper cell, and type 1 T helper cell were higher in PRGcluster A than those in PRGcluster B, while that of CD56dim natural killer cell in PRGcluster A was lower than in PRGcluster B (**Figure 2E**). We performed GSVA enrichment analysis to reveal the regulation pathways in which PRGcluster A was significantly enriched in immune response-related pathways, including NOD-like receptor signaling pathway, B-cell receptor signaling pathway, T-cell receptor signaling pathway, natural killer cell-mediated cytotoxicity, chemokine signaling pathway, primary immunodeficiency, and cytokine receptor interaction (**Figure 2F**). On the basis of the above analysis, PRGcluster A was classified as an immunoinflammatory phenotype, characterized by adaptive immune cell infiltration and immune activation, whereas PRGcluster B was classified as immune-excluded phenotype.

### Generation of gene subtypes based on PRG clusters

**Figure 3:**
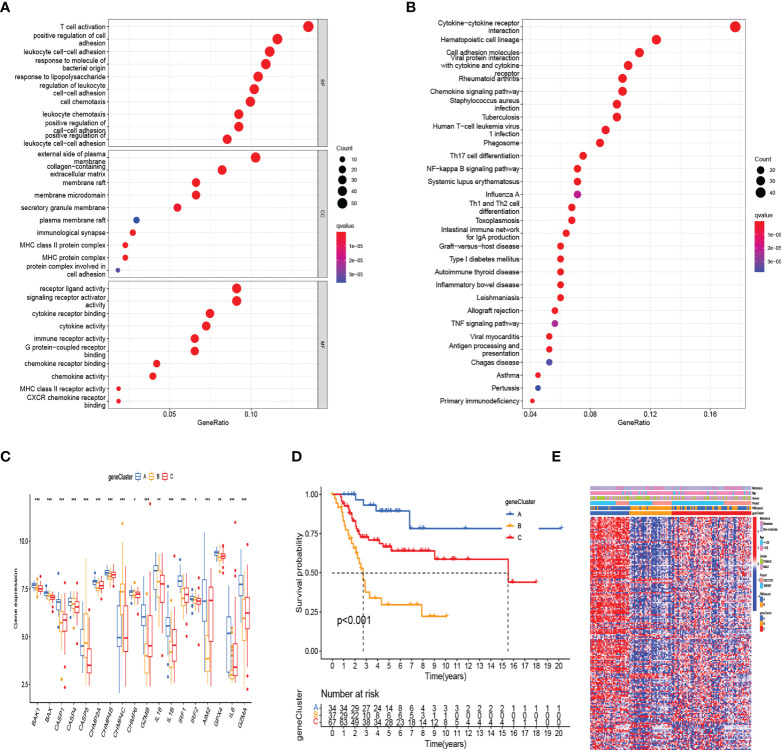
Identification of gene subtypes based on DEGs between two RRG clusters. **(A, B)** The functional enrichment analysis of DEGs among two PRGclusters. **(C)** The differential expressions of 18 PRGs among the three gene subtypes. (***:p value <0.001, **:p value <0.01, *:p value <0.05) **(D)** Kaplan–Meier analysis for overall survival of the three gene subtypes. **(E)** Relationships between clinicopathologic features and the two gene subtypes. DEGs, differentially expressed genes; PRGs, pyroptosis-related genes.

To further define the potential biological function of different pyroptosis clusters, 453 PRGcluster-related DEGs were identified between PRGcluster A and PRGcluster B (**Table S5**). The functional enrichment analysis were performed to indicate that these DEGs were enriched in biological processes of GO and cytokine receptor interaction, cell adhesion molecules, and chemokine signaling pathway of KEGG, which were correlated with immune response regulation (**Figures 3A, B**). After that, to identify the prognostic value of 453 DEGs, a univariate Cox regression analysis was conducted, and 189 prognostic genes were screened out (**Table S6**). Based on 189 prognostic genes, 141 patients with OS were classified into three genomic subtypes using a consensus clustering algorithm to understand the intrinsic regulation mechanism: geneClusters A, B, and C (**Figure S1**). The expressions of PRGs in the three gene clusters were significantly different (**Figure 3C**). The differences were significant in survival time among the three gene clusters (p < 0.001), and the results of the K–M survival curves showed that geneCluster A had the best survival, and geneCluster B was significantly related to poor prognosis (**Figure 3D**). The heatmap shows the correlation of clinical characteristics, pyroptosis clusters, and gene clusters. The different gene expression profiles were observed between geneCluster A, geneCluster B, and geneCluster C (**Figure 3E**).

### Construction and validation of the prognostic PRG_score

**Figure 4:**
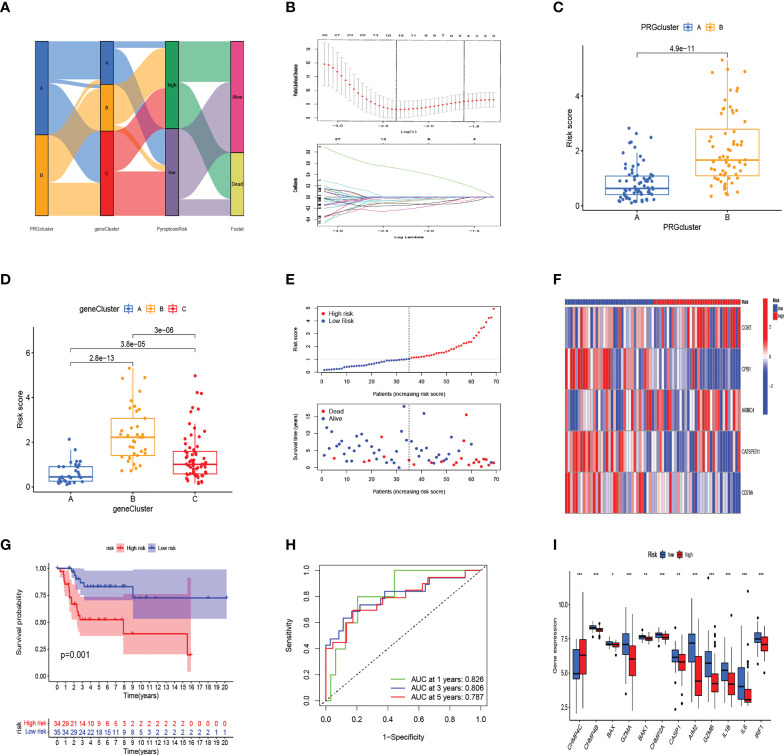
In the training set, generation of PRG_score to predict patient survival. **(A)** Alluvial diagram of pyroptosis-related clusters in groups with different geneClusters, PRG_score, and overall survival. **(B)** The minimal standard was used in the LASSO–Cox model to obtain the value of the super parameter *via* 10-fold cross-validation. **(C)** The differences in PRG_score between PRGclusters. **(D)** The differences in PRG_score between geneClusters. **(E)** Ranked dot and scatter plots showing PRG score distribution and survival status. **(F)** The expression heatmap of the five-gene signature in the training group. **(G)** Kaplan–Meier analysis of the survival between the high- and low-risk groups. **(H)** The prognostic accuracy of the risk scores in the training group was verified by the ROC curve. **(I)** Expression of PRGs in the high- and low-risk groups. (***:p value <0.001,**:p value <0.01,*:p value <0.05) PRG, pyroptosis-related gene; ROC, receiver operating characteristic.

The alluvial diagram illustrates the changes in the attributes of patients in the two pyroptosis clusters, three gene clusters, and two PRG_score groups (**Figure 4A**). We established a pyroptosis-related signature score to quantify each patient based on the 189 prognostic genes, which was named as PRG_score. The patients were randomly divided into training (n = 69) and testing (n = 69) groups using the “caret” package. Next, a signature with seven of the 189 prognosis genes was obtained by application of LASSO–Cox regression with a minimum of lambda value (**Figure 4B**). A stepwise multivariate Cox regression was then performed to analyze seven prognosis genes, finally obtaining five genes (CORT, CPB1, ARMC4, CATSPER1, CD79A; **Table S7**). The outcomes of the multivariate Cox regression analysis showed that PRG_score was constructed as follows: Risk score = (0.601670827227929*expression of CORT) + (-1.39124104164683 *expression of CPB1) + (0.470462955630426*expression of ARMC4) + (-0.762527227347988*expression of CATSPER1) + (-1.10584366215719 *expression of CD79A). PRGcluster A had a lower PRG_score than PRGcluster B, which indicated that a lower PRG_score might be associated with immune inducing function (**Figure 4C**). In addition, a significant difference was represented in PRG_scores among geneClusters. PRG_score was the lowest in geneCluster A (**Figure 4D**). Through the “survminer” program to find the median risk score based on the training group, the patients with PRG_score higher than the median risk score were classified into the low-risk group, whereas those with PRG_score lower than the median risk score were identified into the high-risk group. The survival status plot of the training group revealed that survival times decreased with an increase in PRG_scores (**Figures 4E, F**). The Kaplan–Meier plots show that the overall survival of the high-risk group is significantly shorter than the low-risk group (p = 0.019) (**Figure 4G**). The ROC curves of PRG_score showed that the 1-, 3-, and 5-year survival rates were represented by AUC values of 0.730, 0.878, and 0.867, respectively (**Figure 4H**). For the purpose of validating PRG_score’s stability, the testing group and the all-patient group were used as validation groups. Based on the median risk score in the training cohort, the patients in the testing group and all-patient group were also classified into low- and high-risk groups, respectively. It was shown that the low-risk subgroup represents lower death rates and longer survival times than those in the high-risk subgroup. Kaplan–Meier curve analysis also revealed a significantly better survival in the low-risk group compared to that in the high-risk group. ROC curve analysis showed that PRG_score had relatively high AUC values and predicted the survival of OS patients excellently (**Figure S2**). We also evaluated the correlation between PRGs and our risk model. Fourteen pyroptosis genes were differentially expressed in the high-risk and low-risk groups (**Figure 4I**).

### The clinical prediction and immune infiltration of PRG_score

**Figure 5:**
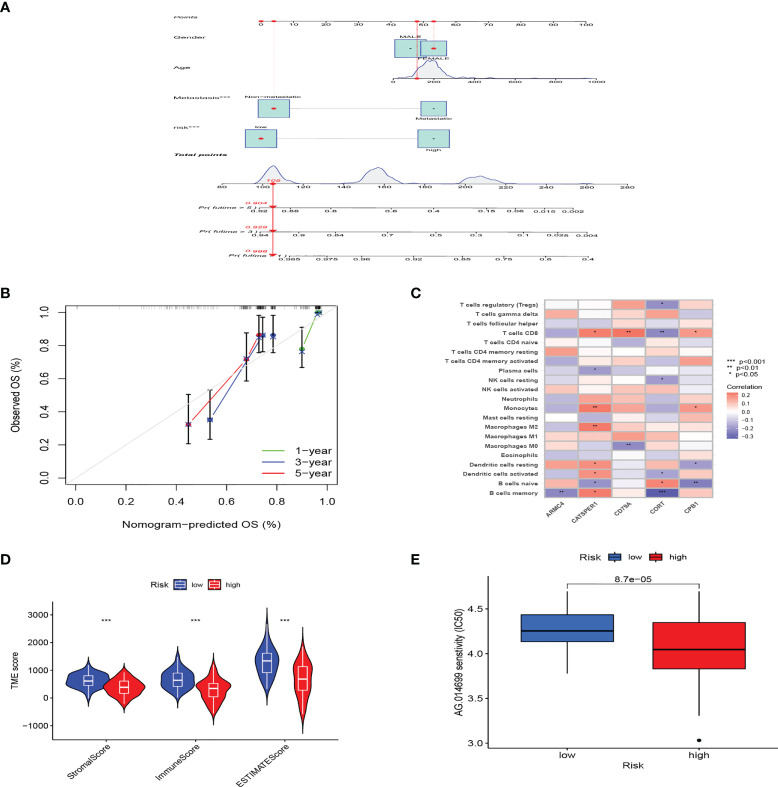
The clinical application value of PRG_score and evaluation of the TME of different subgroups. **(A)** In the training group, nomogram for predicting the 1-, 3-, and 5-year survival of OS patients. **(B)** In the training group, calibration curves of the nomogram for predicting of 1-, 3-, and 5-year overall survival. **(C)** Correlation analysis among the tumor infiltrations of immune cells and five genes in the risk model. **(D)** Correlations between PRG_score and TME scores. (***:p value <0.001). **(E)** PRG score and chemotherapeutic sensitivity relationships. PRG, pyroptosis-related gene; TME, tumor microenvironment; OS, osteosarcoma.

Considering that PRG_score was important in predicting the prognosis of OS patients, a nomogram incorporating the clinicopathological features and PRG_score was constructed to predict the survival rates of OS patients at 1, 3, and 5 years (**Figure 5A**). The predictive nomogram included PRG_score, age, gender, and metastasis. The calibration curves suggested that the predictors had a good predictive value (**Figure 5B**). Next, we investigated whether PRG_score has an instructive significance for immunotherapy. We used the CIBERSORT algorithm to assess the correlation of PRG_score and immune cell infiltration. The scatter diagrams showed that PRG_score was negatively correlated with CD8 + T cells, activated memory CD4 + T cells, monocytes, neutrophils, M2 macrophages, and memory B cells and positively correlated with M0 macrophages and naive B cells (**Figure S3**). We also examined the correlation between the five genes in the proposed model and the proportion of immune cells. We discovered that CD8 + T cells, monocytes, M2 macrophages, memory B cells, M0 macrophages, and naive B cells were mainly correlated with the five genes (**Figure 5C**). The ImmuneScore, StromalScore, and ESTIMATEScore of each of the OS samples were determined using the ESTIMATE algorithm. **Figure 5D** represents that PRG_scores were negatively correlated with the ImmuneScore, StromalScore, and ESTIMATEScore, which indicated that the survival of OS patients is influenced by immune cells and stromal cells. Lastly, we looked at the sensitivity of patients in the low- and high-risk groups to a variety of chemotherapeutic agents presently used to treat OS. Patients with low PRG scores had lower IC50 values for chemotherapeutics such as roscovitine, RDEA119, rapamycin, and shikonin, while patients with high PRG scores had considerably lower IC50 values for axitinib, elesclomol, GW.441756, and thapsigargin (**Figures 5E**, **S4**). These findings demonstrated that PRGs were linked to pharmaceutical sensitivity.

### PRG competing endogenous RNA network construction

**Figure 6:**
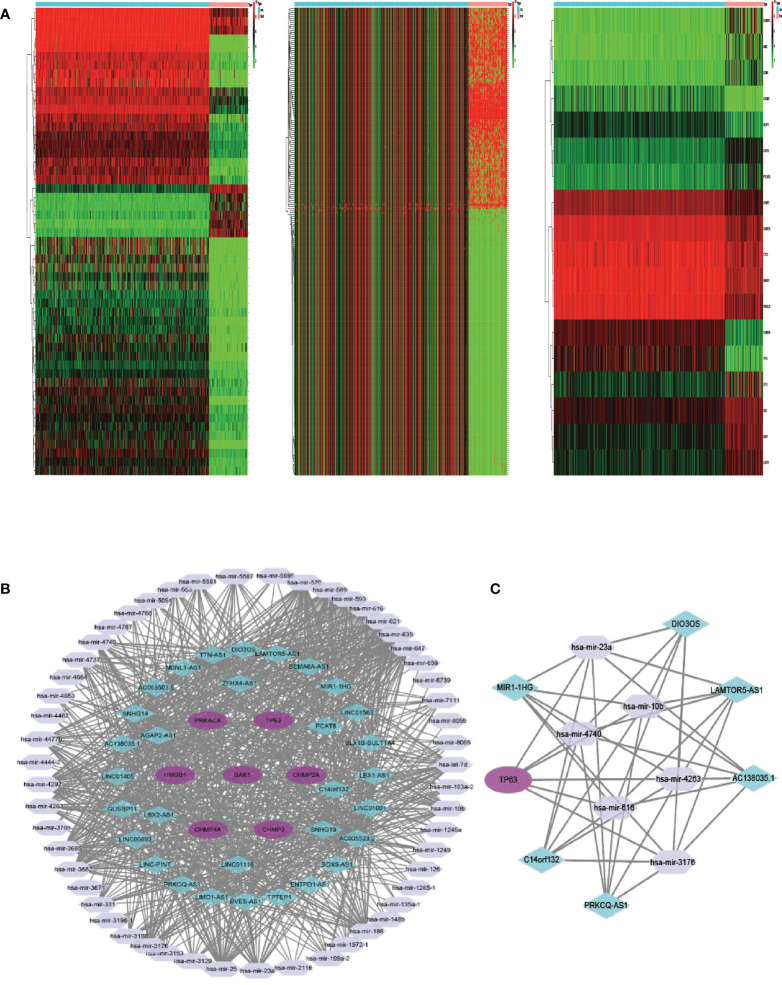
**(A)** Heatmap analysis for differential expressions of mRNAs, lncRNAs, and miRNAs in OS. **(B)** The ceRNA network of seven hub PRGs in OS. **(C)** The network of lncRNA–miRNA–mRNA. OS, osteosarcoma; ceRNA, competing endogenous RNA.

Between 88 OS samples and 396 normal samples, the expression patterns of 52 pyroptosis-related mRNAs, miRNAs, and lncRNAs were determined. A total of 18 pyroptosis-related mRNAs, 53 lncRNAs, and 234 miRNAs were found to be differentially expressed (**Tables S8–S10**). Overexpressed genes included nine pyroptosis-related mRNAs, six lncRNAs, and 100 miRNAs. Nine pyroptosis-related mRNAs, 47 lncRNAs, and 134 miRNAs were all found to be underexpressed. **Figure 6A** depicts the heatmap of clustering analysis of the analyzed RNA. A ceRNA network of the DEls, DEMis, and DEPRMs was constructed using the WGCNA package (**Figure 6B**). We found 31 lncRNA nodes, 53 miRNA nodes, seven mRNA nodes, and 6,153 edges as differentially expressed profiles in the ceRNA network. Using the Cytoscape plug-in MCODE, a cluster with TP63 as the hub gene was extracted from the ceRNA network (**Figure 6C**). Finally, we used the starBase dataset to identify the LAMTOR5-AS1/hsa-miR-23a-3p/TP63 ceRNA regulatory axis.

### Ablation of TP63 and LAMTOR5-AS1 promotes the pyroptosis of OS cells

**Figure 7:**
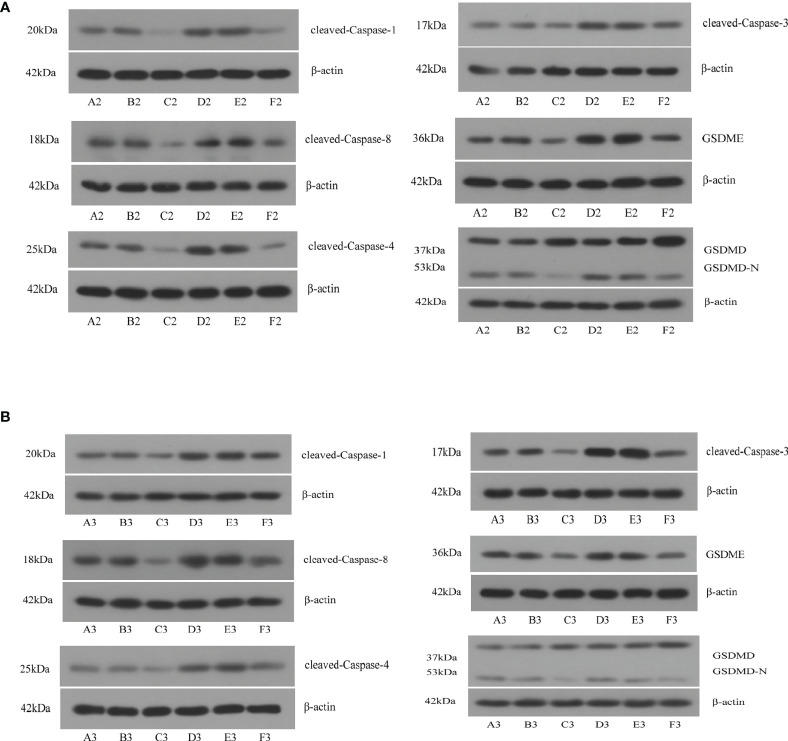
**(A)** After knockdown of TP63, significant decreases were observed on cleaved-Caspase-1, cleaved-Caspase-3, cleaved-Caspase-4, cleaved-Caspase-8, GSDMD, GSDME, and GSDMD-N. A2, B2, C2: 143B cell; D2, E2, F2: U2OS cell. **(B)** After knockdown of LAMTOR5-AS1, significant decreases were observed on cleaved-Caspase-1, cleaved-Caspase-3, cleaved-Caspase-4, cleaved-Caspase-8, GSDMD, GSDME, and GSDMD-N. A2, B2, C2: 143B cell; D2, E2, F2: U2OS cell.

We used shRNA and siRNA separately to silence TP63 and LAMTOR5-AS1 expression, and effective knockdown of TP63 and LAMTOR5-AS1 in both 143B and U2OS cell lines was verified by qRT-PCR (**Table S2**). We observed that abnormal expressions of pyroptosis-related proteins were induced by TP63 knockdown (**Figure 7A**). Cleaved-Caspase-1, which mediates the canonical pathway, and cleaved-Caspase-4, which mediates the non-canonical pathway, both had their expression levels reduced. Caspase-3 and Caspase-8 were previously considered to be marker proteins related to apoptosis, and they can also activate gasdermin proteins under specific induction conditions to regulate the occurrence of pyroptosis (18, 19). When TP63 was knocked down, cleaved-Caspase-3 and cleaved-Caspase-8 also showed decreased expressions. The expressions of GSDMD-N and GSDME, as gasdermin family proteins, were decreased when TP63 was silenced. However, GSDMD was shown to have a negative relationship with TP63. Like the results of TP63 knockdown, the expressions of cleaved-Caspase-1, cleaved-Caspase-3, cleaved-Caspase-4, cleaved-Caspase-8, GSDMD, GSDME, and GSDMD-N showed a significant decrease after LAMTOR5-AS1 knockdown (**Figure 7B**). To summarize, TP63 should be modulated by the LAMTOR5-AS1/hsa-miR-23a-3p ceRNA regulatory network to induce the pyroptosis process of OS cells.

## Discussion

OS is a malignant bone tumor most commonly found in children and adolescents who have a high mortality rate and high morbidity rate. Although chemotherapy and surgery treatments have improved the survival of OS patients, patients with metastases or those who are resistant to chemotherapy necessitate the development of new customized treatment strategies to enhance their prognosis (20). Pyroptosis as an embodiment of programmed cell death is implicated in the potential molecular mechanism of tumors. Numerous studies have indicated that pyroptosis plays a crucial role in various tumors’ growth and metastasis by modulating the TME (21, 22). Therefore, studying the therapeutic benefit and possible molecular mechanism of pyroptosis genes in osteosarcoma is critical. Despite that recent advances had demonstrated the regulatory effect of PRGs on a genetic and transcriptional level for OS, the global alterations in PRGs have not been characterized at CNV and ceRNA in OS.

In this study, using public databases, we determined the expression of 52 pyroptosis-related mRNAs in OS and normal tissues and discovered that most of these mRNAs were expressed differently. Although PRGs had a modest mutation frequency, the bulk of them were disordered in OS patients and were linked to prognosis. The expressions of pyroptosis-related genes were then used to classify individuals with OS. Two distinct pyroptosis patterns of OS patients were identified by the expression of pyroptosis-related genes, which showed that PRGcluster A patients had more advanced survival than PRGcluster B patients. The immune cell infiltration also differed significantly between the two clusters. PRGcluster A was characterized as an immunoinflammatory phenotype, as B cells, CD8+ T cells, immature B cells, macrophages, mast cells, MDSCs, natural killer T cells, and natural killer cells were notably rich in innate immune cell infiltration in PRGcluster A. Moreover, the T-cell receptor signaling pathway, B-cell receptor signaling pathway, NOD-like receptor signaling pathway, and chemokine signaling pathway were all found to be significantly related to immune activation in cluster A. Using the DEGs between the two subtypes of pyroptosis, three gene clusters were identified and proved to be significant in PRGs. As a result, PRGs might be used to predict the clinical prognosis and chemical therapeutic response of OS patients. We developed PRG_score, a reliable and useful prognostic tool, and proved its predictive power. The CNV, TME, prognosis, and drug susceptibility of patients with high-risk and low-risk PRG_scores were significantly different. Then, we created a quantitative nomogram by combining the PRG_score and gender, which improved PRG_score to be better utilized clinically. The predictive model could be used to stratify the OS patients’ prognosis as well as help researchers better understand the disease’s underlying process and provide novel treatment options.

According to various studies, the immune cells and stromal cells in the TME play critical regulatory roles in the OS patients’ prognosis (23, 24). The findings of our study was consistent with the results abovementioned. The stromal score, immune score, and estimate score in the lower PRG_score group were all higher than in the higher PRG_score group, which indicated the TME as an independent risk factor influencing the prognosis of OS. Moreover, the immune microenvironment in the TME could play an important role for OS. For the present study, the relative numbers of immune cells infiltrating tumors varied considerably in two different pyroptosis clusters and two different PRG_score groups. Consequently, this finding suggested that PRGs play an important role in OS immunity regulation. PRGcluster B, which exhibited immune inhibition, had a higher PRG_score, while PRGcluster A, which exhibited immune activation, had a lower PRG_score. geneCluster A was mainly from PRGcluster A, geneCluster B from PRGcluster B, and geneCluster C from PRGcluster A and PRGcluster B, and their PRG_scores were in the following arrangement: geneCluster B > geneCluster C > geneCluster A. This suggested that immunomodulation plays an important role in OS patients’ prognosis.

According to growing evidence, macrophages and CD8+ T lymphocytes play a critical role in OS immune response (25, 26). A lower CD4+/CD8+ ratio in the peripheral blood of OS patients was associated with a greater risk of mortality (27). Anne et al. suggested that CD8+ T lymphocytes were related to a lower risk of OS metastases at the time of diagnosis (25). With a better prognosis, PRGcluster A and low PRG score exhibited increased infiltration of CD8 + T cells, suggesting that they play an antitumor immunology role in OS progression. Increasing data suggest that the immunological context of the osteosarcoma microenvironment is mostly made up of tumor-associated macrophages, with a high ratio of M0 and M2 macrophages (28–30). Unlike macrophages’ tumor-supportive role in many other tumor types, macrophage infiltration was associated with improved survival in OS (31, 32). In high-grade osteosarcoma patients, Buddin et al. showed that CD14-expressed macrophages were related to metastasis suppression and enhanced overall survival (33). However, several studies have shown conflicting results when it comes to the correlation between macrophage phenotypes and clinical prognosis in OS (34, 35). The results of this study indicated that the M1 and M2 macrophage infiltrations in the low PRG score group were significantly higher than those in the high PRG score group. Moreover, the patients with higher M0 and M2 macrophage infiltration had a favorable survival rate. Lastly, we investigated the sensitivity of patients in the low- and high-risk groups to a variety of chemotherapeutic agents presently used to treat OS. It was shown that patients with low PRG scores had lower IC50 values for chemotherapeutics such as roscovitine, RDEA119, rapamycin, and shikonin, while patients with high PRG scores had considerably lower IC50 values for axitinib, elesclomol, GW.441756, and thapsigargin. Using these findings, we would be able to provide our patients with a more accurate targeted therapy.

To find the hub PRG for OS regulation, a ceRNA network was constructed and a potential LAMTOR5-AS1/hsa-miR-23a-3p/TP63 regulatory axis was proposed. The TP63 gene belongs to the tumor-suppressor gene TP53 family, located on chromosome 3q28; it has a high degree of homology with TP53 in sequence and structure, so some of its biological functions are similar to TP53 (36). Sayles et al. demonstrated that TP53 alterations including structural variation (SV) and somatic nucleotide variants (SNVs) are detected in 74% of human osteosarcoma (37). Ito et al. found that 35% of osteosarcoma cases have over three-fold MDM2 amplification (38). Another major inhibitor of TP53 is MDM4. Although it is a homolog of MDM2, MDM4 does not have ubiquitin ligase activity like MDM2. However, MDM4 still binds with TP53 and inhibits TP53 activity (PMID: 30689920, PMCID: PMC6478121, DOI: 10.1093/jmcb/mjz007). Unfortunately, to our understanding, there were no studies discussing about the correlations between TP53 and MDM2 together with MDM4; therefore, the mechanisms and axis between them need to be further investigated. As a pyroptosis hub gene, TP63 may be involved in various aspects in the modulation of pyroptosis in tumors. Celardo et al. (39) overexpressed TP63 in the OS Saos-2 cell line, and the results showed that Caspase-1 expression increased with time in a time-dependent way. Further verification showed that TP63, as a transcription factor, can bind to the promoter of the Caspase-1 gene and promote the transcription of the Caspase-1 gene. Caspase-1 is an important node in the activation of the classical pathway of pyroptosis, and TP63 may promote osteosarcoma pyroptosis by increasing the expression of Caspase-1 (40). In breast cancer, TP63 induced the expression of GSDME *via* binding a specific site in GSDME (41). The findings of this study are consistent with the above conclusions. After silencing of the gene TP63 by siRNA transfection in OS cells, the protein levels of cleaved-Caspase-1 and GSDME were downregulated when measured by WB, and other pyroptosis marker proteins including cleaved-Caspase-3, cleaved-Caspase-4, cleaved-Caspase-8, and GSDMD-N were also downregulated. This indicated that TP63 could activate cell pyroptosis in OS through multiple pathways including canonical (Caspase-1 mediated) and non-canonical (Caspase-4 mediated) pathways. Moreover, we used the starBase v2.0 database to predict that LAMTOR5-AS1 regulates the expression of TP63 in OS through the ceRNA mechanism in combination with hsa-miR-23a-3p. Pu et al. (42) demonstrated that LAMTOR5-AS1 reduces OS cell growth and multidrug resistance in a considerable way. In this study, LAMTOR5-AS1 knockdown decreased the expression of cleaved-Caspase-1, cleaved-Caspase-3, cleaved-Caspase-4, cleaved-Caspase-8, GSDME, and GSDMD-N in OS cells, which demonstrated that the type and expression trend of pyroptosis marker proteins regulated by LAMTOR5-AS1 was consistent with those regulated by TP63. This could prove that TP63 as hub pyroptosis gene could be modulated by the LAMTOR5-AS1/hsa-miR-23a-3p ceRNA regulatory network.

There were various flaws in this research. To begin, all studies were based exclusively on data from public sources, and clinical samples were not collected. As a consequence, there may have been an inherent bias in selection of cases that affected the study findings. To corroborate our results, large-scale prospective investigations as well as more *in vivo* and *in vitro* experimental research are required. Furthermore, the LAMTOR5-AS1/hsa-miR-23a-3p/TP63 ceRNA network lacked validation by using the luciferase reporter system to confirm that hsa-miR-23a-3p was the miRNA sponged by LAMTOR5-AS1.

## Conclusions

Based on our thorough investigation of PRGs, we found a complex regulatory system through which they influence the tumor-immune-stroma environment, clinicopathological characteristics, and prognosis. Meanwhile, a ceRNA network was built to find a LAMTOR5-AS1/hsa-miR-23a-3p/TP63 regulatory axis. We also further looked at PRGs’ therapeutic potential in targeted therapy and immunotherapy. These results emphasized PRGs’ critical clinical significance and provide fresh ideas for directing individualized chemotherapy and immunotherapy for OS patients.

## Data availability statement

The original contributions presented in the study are included in the article/**Supplementary Material**. Further inquiries can be directed to the corresponding author.

## Author contributions

The study was designed and conceptualized by JH, YH, SD, and HW. JH, YH, SD, and SL curated the data. The statistical analysis was carried out by JH, and YH. The manuscript was written by JH and YH. The study was overseen by HW. We all revised the manuscript, read the submitted version, and agreed with it.

## Funding

This work was funded by the National Natural Science Foundation of China (grant number: 31971275).

## Conflict of interest

The authors declare that the research was conducted in the absence of any commercial or financial relationships that could be construed as a potential conflict of interest.

## Publisher’s note

All claims expressed in this article are solely those of the authors and do not necessarily represent those of their affiliated organizations, or those of the publisher, the editors and the reviewers. Any product that may be evaluated in this article, or claim that may be made by its manufacturer, is not guaranteed or endorsed by the publisher.

## Supplementary material

Supplementary Figure 1 Consensus matrix heatmap defining two clusters (k = 3) in TARGET and GSE21257 cohort based on DEGs expression.

Supplementary Figure 2
**(A)** PRG score distribution and survival status in ranked dot and scatter plots in the testing group. **(B)** The expression heatmap of 5 gene signature in the testing group. **(C)** Kaplan–Meier analysis of the survival between the high- and low-risk groups in the testing group. **(D)** The prognostic accuracy of the risk scores in the testing group was verified by ROC curve. **(E)** PRG score distribution and survival status in ranked dot and scatter plots in the all group. **(F)** The expression heatmap of 5 gene signature in the all group. **(G)** Kaplan–Meier analysis of the survival between the high- and low-risk groups in the all group. **(H)** The prognostic accuracy of the risk scores in the all group was verified by ROC curve.

Supplementary Figure 3
**(A–H)** Correlations between PRG_score and immune cell types.

Supplementary Figure 4 Relationships between PRG_score and chemotherapeutic sensitivity.
