# Supplementary material for: The analysis of the pyroptosis-related genes and hub gene TP63 ceRNA axis in osteosarcoma
Source: Front Immunol. 2022 Nov 1;13:974916. doi: 10.3389/fimmu.2022.974916 (PMC9664215; doi:10.3389/fimmu.2022.974916)
Supplement: Supplementary file 1 [file Table_1.docx]

Gene Type

BAK1 pyroptosis

BAX pyroptosis

CASP1 pyroptosis

CASP3 pyroptosis

CASP4 pyroptosis

CASP5 pyroptosis

CHMP2A pyroptosis

CHMP2B pyroptosis

CHMP3 pyroptosis

CHMP4A pyroptosis

CHMP4B pyroptosis

CHMP4C pyroptosis

CHMP6 pyroptosis

CHMP7 pyroptosis

CYCS pyroptosis

ELANE pyroptosis

GSDMD pyroptosis

GSDME pyroptosis

GZMB pyroptosis

HMGB1 pyroptosis

IL18 pyroptosis

IL1A pyroptosis

IL1B pyroptosis

IRF1 pyroptosis

IRF2 pyroptosis

TP53 pyroptosis

TP63 pyroptosis

AIM2 pyroptosis

CASP6 pyroptosis

CASP8 pyroptosis

CASP9 pyroptosis

GPX4 pyroptosis

GSDMA pyroptosis

GSDMB pyroptosis

GSDMC pyroptosis

IL6 pyroptosis

NLRC4 pyroptosis

NLRP1 pyroptosis

NLRP2 pyroptosis

NLRP3 pyroptosis

NLRP6 pyroptosis

NLRP7 pyroptosis

NOD1 pyroptosis

NOD2 pyroptosis

PJVK pyroptosis

PLCG1 pyroptosis

PRKACA pyroptosis

PYCARD pyroptosis

SCAF11 pyroptosis

TIRAP pyroptosis

TNF pyroptosis

GZMA pyroptosis
