# Supplementary figures and images for: The analysis of the pyroptosis-related genes and hub gene TP63 ceRNA axis in osteosarcoma
Source: Front Immunol. 2022 Nov 1;13:974916. doi: 10.3389/fimmu.2022.974916 (PMC9664215; doi:10.3389/fimmu.2022.974916)

A

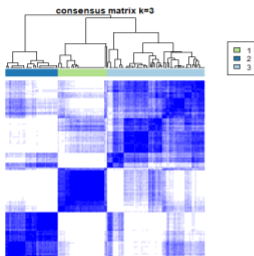

B

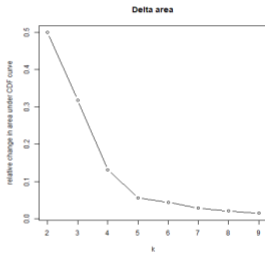

Supplement: Supplementary Figure 1 — Consensus matrix heatmap defining two clusters (k = 3) in TARGET and GSE21257 cohort based on DEGs expression. [file DataSheet_1.pdf]

A

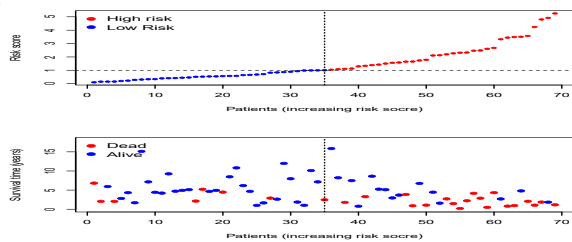

B

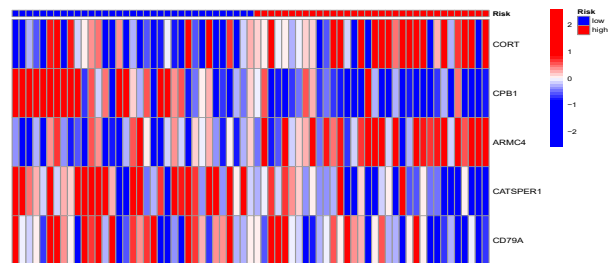

C

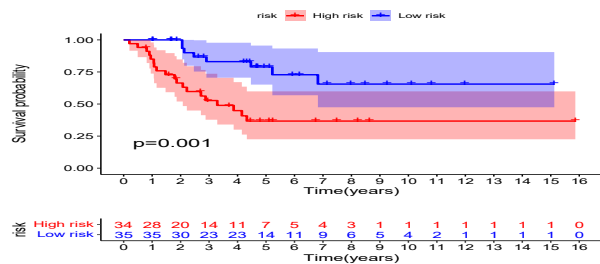

D

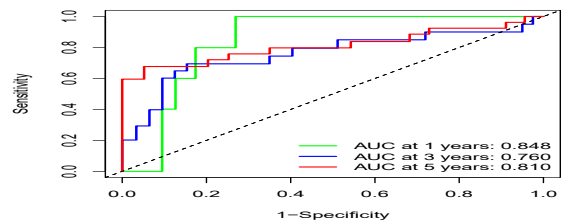

E

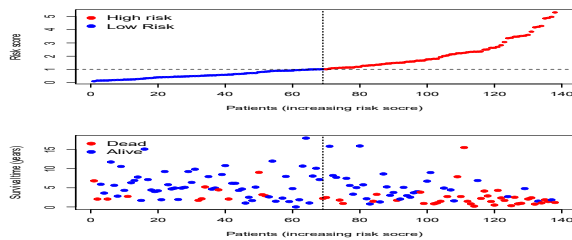

F

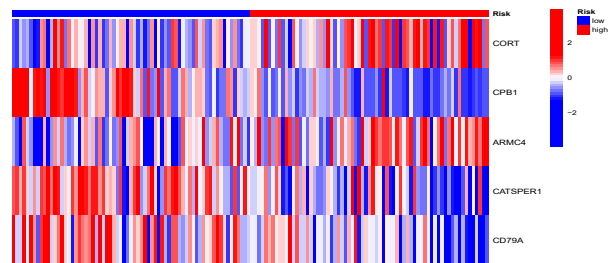

G

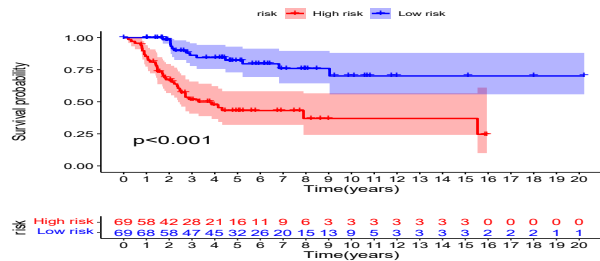

H

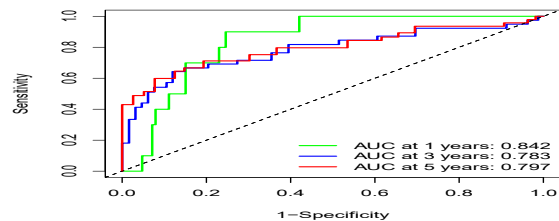

Supplement: Supplementary Figure 2 — (A) PRG score distribution and survival status in ranked dot and scatter plots in the testing group. (B) The expression heatmap of 5 gene signature in the testing group. (C) Kaplan–Meier analysis of the survival between the high- and low-risk groups in the testing group. (D) The prognostic accuracy of the risk scores in the testing group was verified by ROC curve. (E) PRG score distribution and survival status in ranked dot and scatter plots in the all group. (F) The expression heatmap of 5 gene signature in the all group. (G) Kaplan–Meier analysis of the survival between the high- and low-risk groups in the all group. (H) The prognostic accuracy of the risk scores in the all group was verified by ROC curve. [file DataSheet_2.pdf]

A

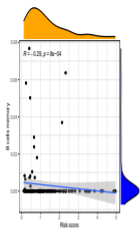

B

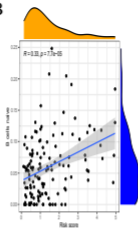

C

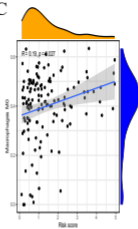

D

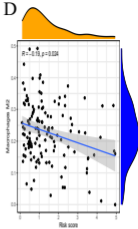

E

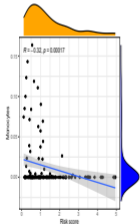

F

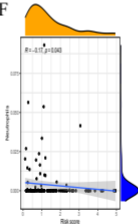

G

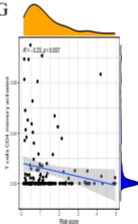

H

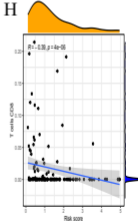

Supplement: Supplementary Figure 3 — (A–H) Correlations between PRG_score and immune cell types. [file DataSheet_3.pdf]

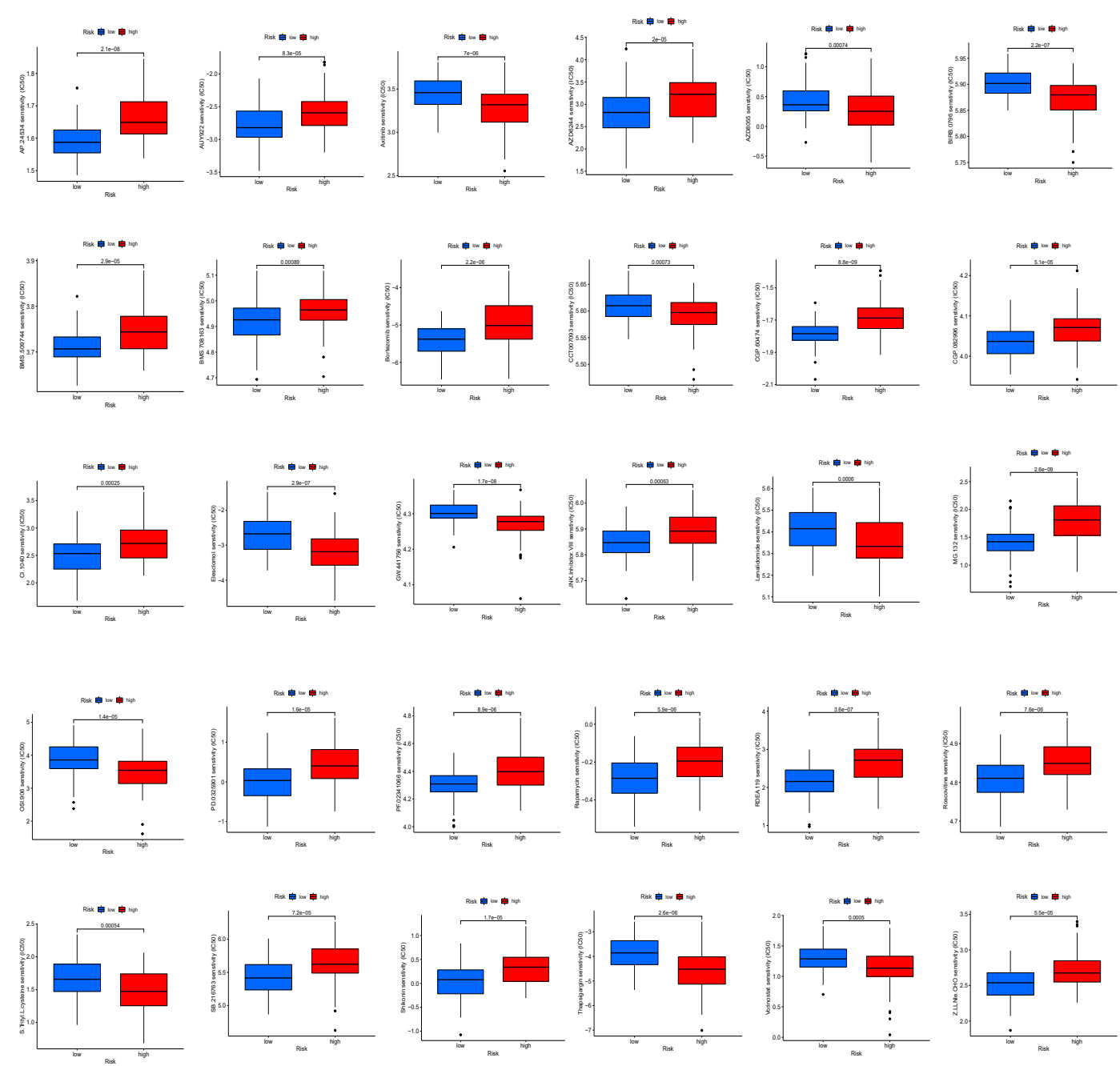

Supplement: Supplementary Figure 4 — Relationships between PRG_score and chemotherapeutic sensitivity. [file DataSheet_4.pdf]
